# Supplementary material for: Epigenetic age acceleration is associated with oligodendrocyte proportions in MSA and control brain tissue
Source: Neuropathol Appl Neurobiol. 2023 Jan 11;49(1):e12872. doi: 10.1111/nan.12872 (PMC10107510; doi:10.1111/nan.12872)
Supplement: Supplementary file 1 — Figure S1 Association between age acceleration and neuronal proportions for DNAmClockCortical and DNAmClockMulti in the different brain regions. (a‐d) Age acceleration residuals (y‐axis) versus neuronal proportions (x‐axis) for DNAmClockCortical and (e‐h) Age acceleration residuals (y‐axis) versus neuronal proportions (x‐axis) for DNAmClockMulti in the different brain regions. Age acceleration residuals were obtained by regressing DNA methylation age against confounding factors, including chronological age; neuronal proportions were obtained using a DNA methylation‐based cell‐type deconvolution algorithm. The correlation coefficient and p‐values shown were calculated using Pearson correlation. CC – control cerebellum (WM); MC – multiple system atrophy (MSA) cerebellum (WM); CF – control frontal lobe (WM); MF – MSA frontal lobe (WM); CO – control occipital lobe (WM); MO – MSA occipital lobe (WM); PFC – prefrontal cortex (GM + WM), CpC – Corpus callosum; WM – white matter; GM – grey matter. Figure S2 Association between age acceleration and oligodendrocyte proportions for the multiple system atrophy (MSA) cases and control groups for DNAmClockCortical and DNAmClockMulti in the different brain regions (a‐g) Age acceleration residuals (y‐axis) versus oligodendrocyte (SOX10+) proportions (x‐axis) for the DNAmClockCortical and (h‐n) Age acceleration residuals (y‐axis) versus oligodendrocyte (SOX10+) proportions (x‐axis) for the DNAmClockMulti in the different brain regions. Age acceleration residuals were obtained by regressing DNA methylation age against confounding factors, including chronological age; oligodendrocyte proportions were obtained using a DNA methylation‐based cell‐type deconvolution algorithm. The correlation coefficient and p‐values shown were calculated using Pearson correlation. CC – control cerebellum (WM); MC – MSA cerebellum (WM); CF – control frontal lobe (WM); MF – MSA frontal lobe (WM); CO – control occipital lobe (WM); MO – MSA occipital lobe (WM [file NAN-49-0-s001.pdf]

Epigenetic age acceleration is associated with oligodendrocyte proportions in MSA and control brain tissue

Megha Murthy[1,2], Gemma Shireby[3], Yasuo Miki[1,4], Emmanuelle Viré[5], Tammaryn Lashley[1,6], Thomas T. Warner[1,2,7], Jonathan Mill[3], Conceição Bettencourt[1,6*]

[1]Queen Square Brain Bank, UCL Queen Square Institute of Neurology, University College London, UK

[2]Department of Clinical and Movement Neurosciences, UCL Queen Square Institute of Neurology, London, UK.

[3]University of Exeter Medical School, University of Exeter, UK

[4]Department of Neuropathology, Institute of Brain Science, Hirosaki University Graduate School of Medicine, Hirosaki, Japan.

[5]UCL Institute of Prion Diseases, MRC Prion Unit at UCL, University College London, London, UK.

[6]Department of Neurodegenerative Disease, UCL Queen Square Institute of Neurology, London, UK.

[7]Reta Lila Weston Institute, UCL Queen Square Institute of Neurology, London, UK.

***Corresponding Author:**

Conceição Bettencourt, PhD

Department of Clinical and Movement Neurosciences

UCL Queen Square Institute of Neurology

Queen Square Brain Bank for Neurological Disorders

1 Wakefield Street

London WC1N 1PJ

United Kingdom

Email: c.bettencourt@ucl.ac.uk

Supplementary Information

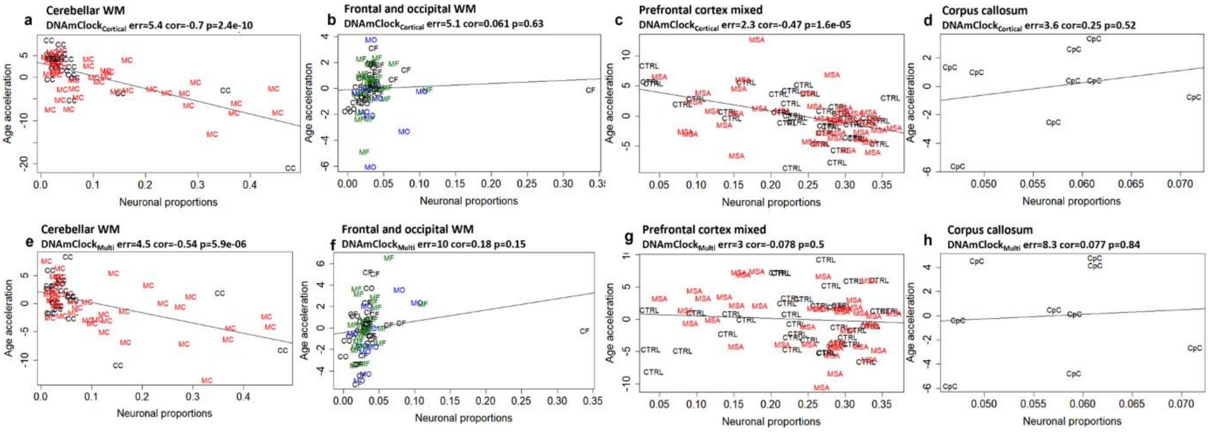

Figure S1. Association between age acceleration and neuronal proportions for DNAmClock$_{\text{Cortical}}$ and DNAmClock$_{\text{Multi}}$ in the different brain regions. (a-d) Age acceleration residuals (y-axis) versus neuronal proportions (x-axis) for DNAmClock$_{\text{Cortical}}$ and **(e-h)** Age acceleration residuals (y-axis) versus neuronal proportions (x-axis) for DNAmClock$_{\text{Multi}}$ in the different brain regions. Age acceleration residuals were obtained by regressing DNA methylation age against confounding factors, including chronological age; neuronal proportions were obtained using a DNA methylation-based cell-type deconvolution algorithm. The correlation coefficient and p-values shown were calculated using Pearson correlation. CC – control cerebellum (WM); MC – multiple system atrophy (MSA) cerebellum (WM); CF – control frontal lobe (WM); MF – MSA frontal lobe (WM); CO – control occipital lobe (WM); MO – MSA occipital lobe (WM); PFC – prefrontal cortex (GM+WM), CpC – Corpus callosum; WM – white matter; GM – grey matter.

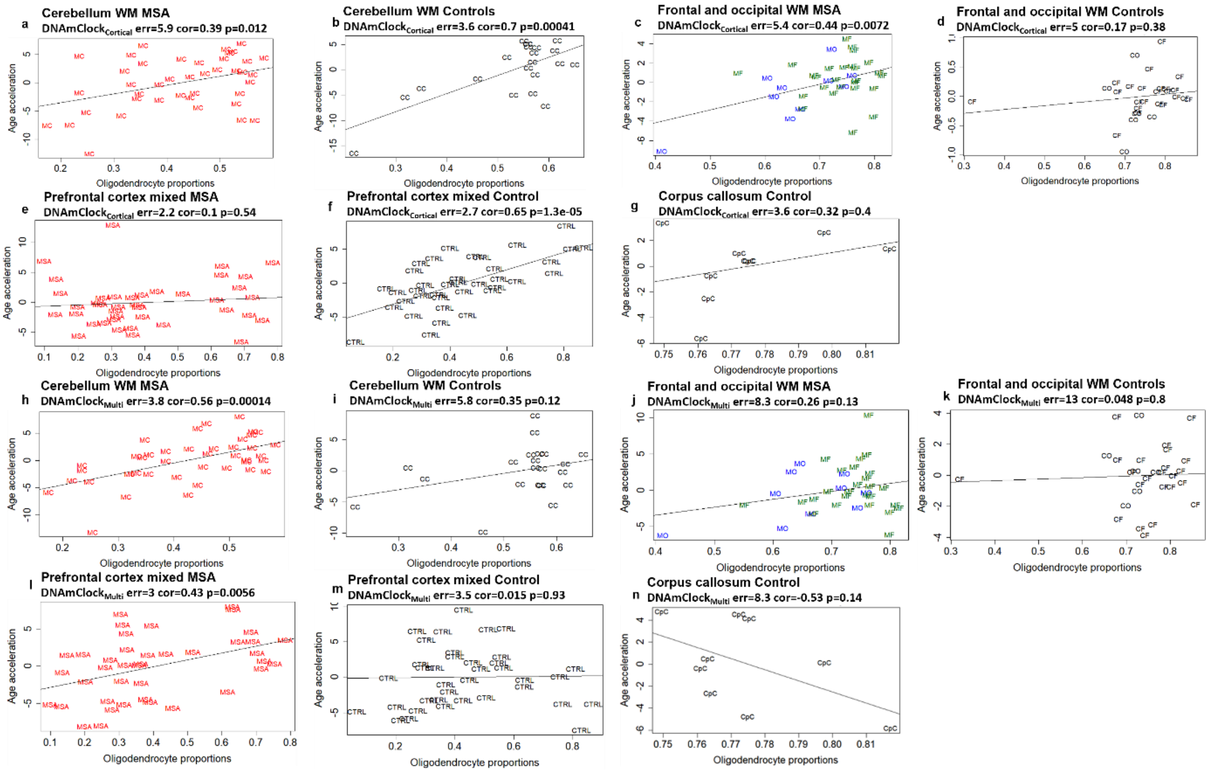

Figure S2. Association between age acceleration and oligodendrocyte proportions for the multiple system atrophy (MSA) cases and control groups for DNAmClock$_{Cortical}$ and DNAmClock$_{Multi}$ in the different brain regions (a-g) Age acceleration residuals (y-axis) versus oligodendrocyte (SOX10+) proportions (x-axis) for the DNAmClock$_{Cortical}$ and **(h-n)** Age acceleration residuals (y-axis) versus oligodendrocyte (SOX10+) proportions (x-axis) for the DNAmClock$_{Multi}$ in the different brain regions. Age acceleration residuals were obtained by regressing DNA methylation age against confounding factors, including chronological age; oligodendrocyte proportions were obtained using a DNA methylation-based cell-type deconvolution algorithm. The correlation coefficient and p-values shown were calculated using Pearson correlation. CC – control cerebellum (WM); MC – MSA cerebellum (WM); CF – control frontal lobe (WM); MF – MSA frontal lobe (WM); CO – control occipital lobe (WM); MO – MSA occipital lobe (WM); PFC - prefrontal cortex (GM+WM); CpC – Corpus callosum; WM – white matter; GM – grey matter.

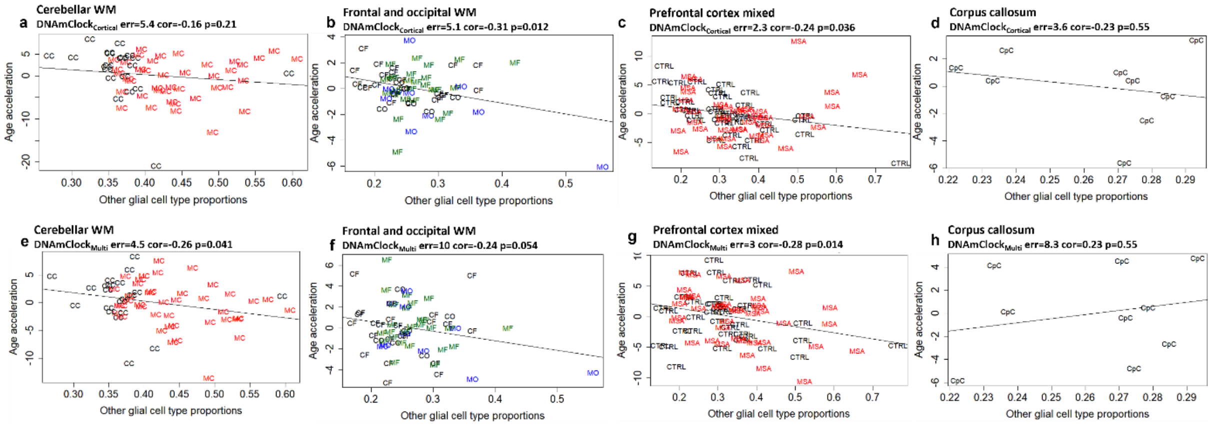

Figure S3. Association between age acceleration and other glial cell type (NeuN-/SOX10-) populations for DNAmClock$_{Cortical}$ and DNAmClock$_{Multi}$ clocks in the different brain regions. (a-d) Age acceleration residuals (y-axis) versus other brain cell type (NeuN-/SOX10-) proportions (x-axis) for the DNAmClock$_{Cortical}$ and **(e-h)** Age acceleration residuals (y-axis) versus other brain cell type (NeuN-/SOX10-) proportions (x-axis) for the DNAmClock$_{Multi}$ in the different brain regions. Age acceleration residuals were obtained by regressing DNA methylation age against confounding factors, including chronological age; NeuN-/SOX10- proportions were obtained using a DNA methylation-based cell-type deconvolution algorithm. The correlation coefficient and p-values shown were calculated using Pearson correlation. CC – control cerebellum (WM); MC – multiple system atrophy (MSA) cerebellum (WM); CF – control frontal lobe (WM); MF – MSA frontal lobe (WM); CO – control occipital lobe (WM); MO – MSA occipital lobe (WM); PFC - prefrontal cortex (GM+WM), CpC – Corpus callosum; WM – white matter; GM – grey matter.

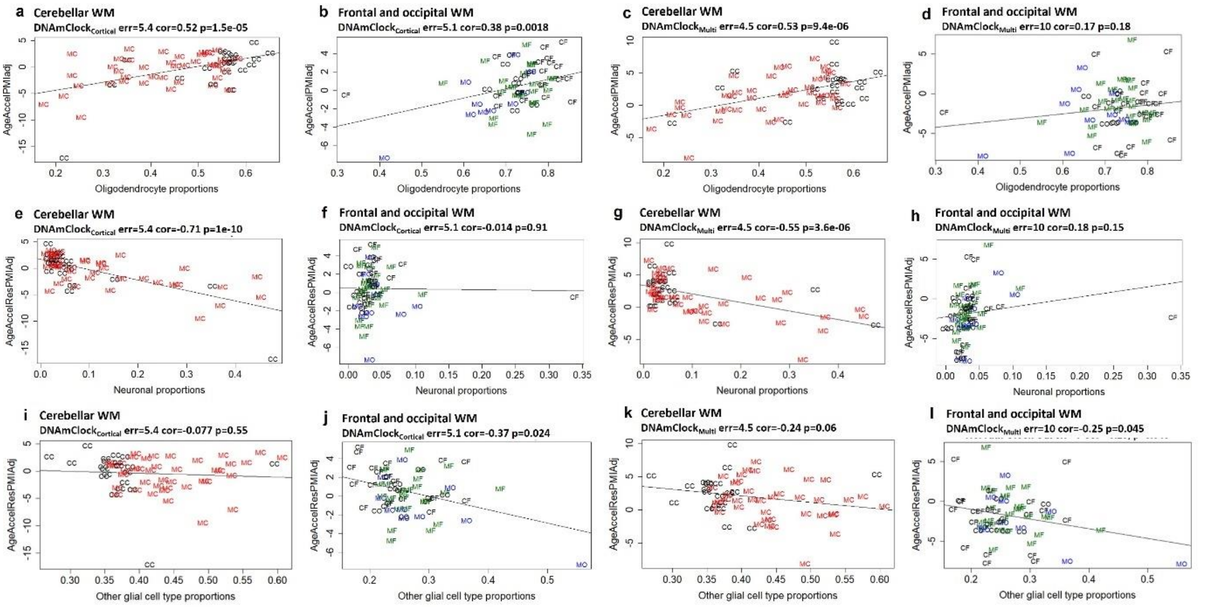

Figure S4. Association between age acceleration and the different cell type proportions for DNAmClock$_{Cortical}$ and DNAmClock$_{Multi}$ in the different brain regions after including post-mortem interval (PMI) as a covariate in cohort 1 regression models. (a-d) Age acceleration residuals (y-axis) versus oligodendrocyte (NeuN-/SOX10+) proportions (x-axis) for both clocks; **(e-h)** age acceleration residuals (y-axis) versus neuronal (NeuN+/SOX10-) proportions (x-axis) for both clocks; **(i-l)** age acceleration residuals (y-axis) versus other glial cell type (NeuN-/SOX10-) proportions (x-axis) for both clocks. Age acceleration residuals were obtained by regressing DNA methylation age against confounding factors, including chronological age; cell proportions were obtained using a DNA methylation-based cell-type deconvolution algorithm. The correlation coefficient and p-values shown were calculated using Pearson correlation. CC – control cerebellum (WM); MC – multiple system atrophy (MSA) cerebellum (WM); CF – control frontal lobe (WM); MF – MSA frontal lobe (WM); CO – control occipital lobe (WM); MO – MSA occipital lobe (WM); WM – white matter.

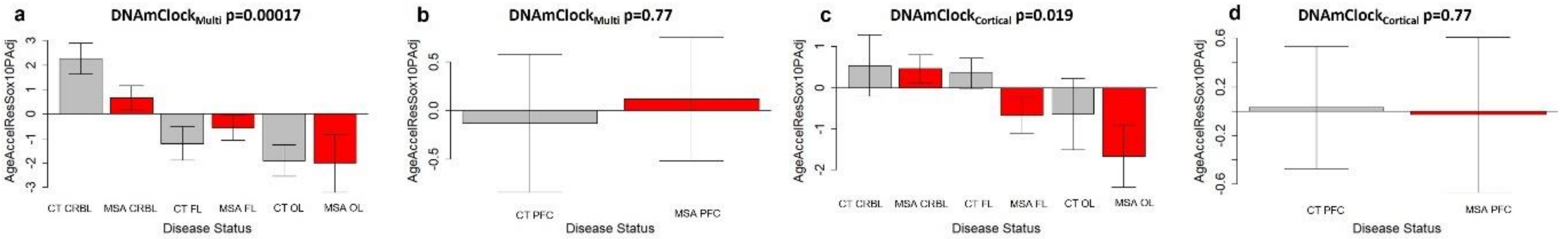

Figure S5. Age acceleration estimation after adjusting for oligodendrocyte proportions for DNAmClock$_{Cortical}$ and DNAmClock$_{Multi}$ in the different brain regions (a-d) Acceleration residual after adjusting for chronological age and oligodendrocyte proportions for the DNAmClock$_{Multi}$ (a - cohort 1; b - cohort 2) and DNAmClock$_{Cortical}$ (c - cohort 1; d - cohort 2) in the different brain regions. The p-values for across group comparisons were calculated using the Kruskal-Wallis test and p-values for pairwise analysis between MSA and controls for each brain region were calculated using the Wilcoxon's test with Benjamini-Hochberg correction for multiple testing. CT CRBL – control cerebellum (WM); MSA CRBL – multiple system atrophy (MSA) cerebellum (WM); CT FL – control frontal lobe (WM); MSA FL – MSA frontal lobe (WM); CT OL – control occipital lobe (WM); MSA OL – MSA occipital lobe (WM); CT PFC – control prefrontal cortex (GM+WM); MSA PFC – MSA prefrontal cortex (GM+WM); WM – white matter; GM – grey matter.

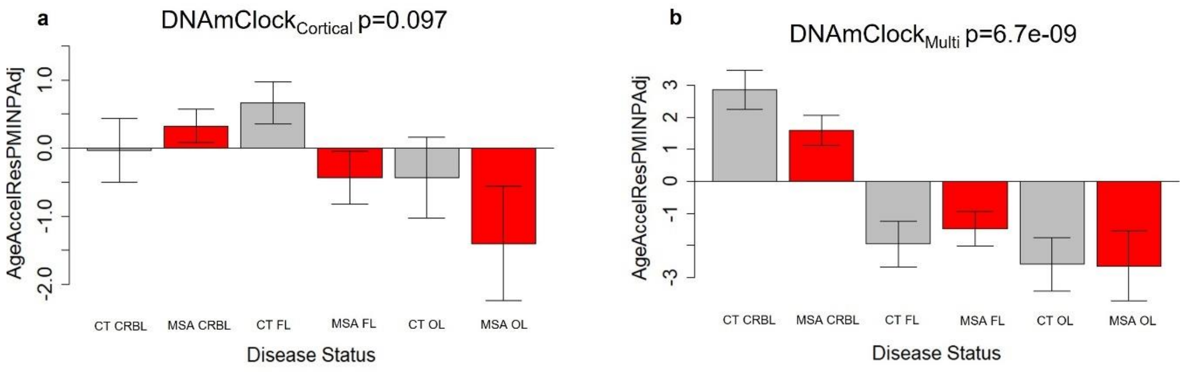

Figure S6. Age acceleration estimation for cohort 1 in the different brain regions for (a) DNAmClock$_{Cortical}$ and (b) DNAmClock$_{Multi}$ after adjusting for post-mortem interval (PMI) (Chronological age, neuronal proportions, replicate individuals and PMI were included in the regression model). CT CRBL - control cerebellum (WM); MSA CRBL – multiple system atrophy (MSA) cerebellum (WM); CT FL – control frontal lobe (WM); MSA FL – MSA frontal lobe (WM); CT OL – control occipital lobe (WM); MSA OL – MSA occipital lobe (WM); CT PFC – control prefrontal cortex (GM+WM); MSA PFC – MSA prefrontal cortex (GM+WM); WM – white matter; GM – grey matter. The p-values for across group comparisons were calculated using the Kruskal-Wallis test and p-values for pairwise analysis between MSA and controls for each brain region were calculated using the Wilcoxon's test with Benjamini-Hochberg correction for multiple testing.

Table S1. Cohort demographics.

Tissue/Region	Controls					MSA				
	No. of samples			Mean Chronological age ±SD		No. of samples			Mean Chronological age ±SD	
**Cohort 1**	**N**	**Females (%)**	**Males (%)**	**Females**	**Males**	**N**	**Females (%)**	**Males (%)**	**Females**	**Males**
Cerebellum (WM)	21	47.6	52.4	81.1±9	79.55±9	41	48.8	51.2	64.2±7	64.48±8
Frontal lobe (WM)	23	47.8	52.2	76±10	76.58±7	26	50	50	65.85±4	67.08±8
Occipital lobe (WM)	6	33.3	66.7	95±5	83.75±8	10	50	50	64.6±3	63.4±8
**Cohort 2**										
Prefrontal cortex (GM+WM)	37	48.6	51.4	72.78±9	73.16±11	40	56.1	41.5	64.91±5	67.47±6
**Cohort 3**										
Corpus callosum	9	22.2	77.8	58±9	61.43±13	-	-	-	-	-
**Cohort 4**										
Sox10+ nuclei	15	53.3	46.7	77.88±12	81.86±8	-	-	-	-	-

MSA - multiple system atrophy; WM - white matter; GM+WM - Mix of grey and white matter; SD - Standard deviation

Table S2. Difference in median absolute deviation between controls and MSA for $DNAmClock_{Multi}$ and $DNAmClock_{Cortical}$.

Region	$DNAmClock_{Cortical}$			$DNAmClock_{Multi}$		
	**CTRL**	**MSA**	**p value (two.sided)**	**CTRL**	**MSA**	**p value (two.sided)**
**Cohort 1**						
Cerebellum (WM)	3.6	5.9	0.158	5.8	3.8	0.014
Frontal lobe (WM)	5.1	5.4	0.912	12	8.3	0.016
Occipital lobe (WM)	2.6	5.3	0.626	16	8.7	0.008
**Cohort 2**						
Prefrontal cortex (GM+WM)	2.7	3.5	0.614	2.2	3	0.795
**Total**			**0.222**			**0.011**

CTRL - control; MSA - multiple system atrophy; WM - white matter; GM+WM - Mix of grey and white matter; the p-values were calculated using two-sided Wilcoxon rank sum test with continuity correction.

Table S3. Mean chronological and DNAm ages for $DNAmClock_{Multi}$ and $DNAmClock_{Cortical}$ for all brain regions of control and MSA samples.

Tissue/Region	N		Mean chronological age		Mean DNAm age			
					$DNAmClock_{Cortical}$		$DNAmClock_{Multi}$	
	CTRL	MSA	CTRL	MSA	CTRL	MSA	CTRL	MSA
**Cohort 1**								
Cerebellum (WM)	21	41	80.28	64.34	81.1	68.9	72.53	60.26
Frontal lobe (WM)	23	26	76.3	66.46	80.3	71.8	64.06	58.3
Occipital lobe (WM)	6	10	87.5	64	84.2	66.98	69.8	54.9
**Cohort 2**								
Prefrontal cortex (GM+WM)	37	41	72.97	66	73.26	66.66	71.27	66.41
**Cohort 3**								
Corpus callosum	9		60.66		57.03		53.17	
**Cohort 4**								
SOX10+ nuclei	15		79.73		90.11		53.74	

CTRL - control; MSA - multiple system atrophy; WM - white matter; GM+WM - Mix of grey and white matter
